# Children and Cinemas

**Published:** 1920-01-24

**Authors:** 


### CHILDREN AND CINEMAS.

THE important question of the effects of cinematograph entertainments on the health of children is again receiving attention, and the London County Council have under consideration especially the question of eye strain. There is evidence of special cases of eye strain as a direct result of visits to cinematograph halls, and perhaps this evil effect is one of the most important. There is also the question of the high degree of nervousness which is produced in some children by attendance at these shows, and this too demands very close and careful attention. With special regard to the question of eye strain, it is satisfactory to note that a special committee has been formed, composed of ophthalmologists and physiologists, as well as representatives of the cinematograph industry, to investigate the subject. It is well that the attention of medical men is thus being drawn to this important consideration, for in view of the proposed increase in the use of the cinematograph for educational purposes, it is wise to weigh carefully its disadvantages.

The eyes of the child are subjected during a cinematograph performance to the most trying exercises. There is, first, a great amount of muscular work to do, involving great fatigue for the eye; there is also the glare and flickering, necessitating rapid change of accommodation, which again is productive of much ocular fatigue.

There is no doubt that the performances should be much shorter, so that the child has a better chance of recovering from the great eye strain to which he has been subjected while in the theatre or hall.

Following this important question of eye strain, next in importance comes the effect on the nervous system, especially in susceptible children, and it cannot be denied that this may be most serious. The definite impression produced from seeing certain excitable pictures cannot fail to have a reactive effect, so that the child reconstructs the drama or story, whatever it be, in its sleep or waking hours, thus producing the restless energy so harmful to a child, whose mind should always be peaceful at these times.

Other points against the constant visiting of cinematograph shows, especially at this season of the year, are the crowded and stuffy atmosphere, so productive of colds and catarrhs, and the frequent excess of tobacco-smoke, which acts as a marked irritant to children's sensitive mucous membranes.

From an educational point of view, there is much to be said for the cinematograph, but medically there is no doubt that parents and others in authority should exercise great moderation in the frequency with which they allow their children to attend these entertainments; and the nervous, excitable children, as well as those with defective vision, should have their visits made few and far between.

